# Clinical characteristics of neonatal fulminant necrotizing enterocolitis in a tertiary Children's hospital in the last 10 years

**DOI:** 10.1371/journal.pone.0224880

**Published:** 2019-11-08

**Authors:** Lu Lin, Xuhua Xia, Wei Liu, Yongming Wang, Ziyu Hua

**Affiliations:** 1 Department of Neonatology, Children’s Hospital of Chongqing Medical University, Chongqing, P.R China; 2 National Clinical Research Center for Child Health and Disorders, Chongqing, P.R China; 3 China International Science and Technology Cooperation base of Child development and Critical Disorders, Chongqing, P.R China; 4 Department of Gastrointestinal and Neonatal Surgery, Children’s Hospital of Chongqing Medical University, Chongqing, P.R China; 5 Chongqing Key Laboratory of Child Health and Nutrition, Chongqing, P.R China; 6 Ministry of Education Key Laboratory of Child Development and Disorders, Chongqing, P.R China; KU Leuven, BELGIUM

## Abstract

The aim of this retrospective study was to explore the risk factors and clinical characteristics related to neonatal fulminant necrotizing enterocolitis (NEC). From 1 November 2007 to 31 October 2017, 352 neonates who were diagnosed with NEC (Bell stage ≥ΠB) and admitted to the Children’s Hospital of Chongqing Medical University were enrolled. Among these patients, 112 (31.82%) cases fulfilled the definition of fulminant NEC, and 62.5% (70/112) of fulminant cases presented a poor prognosis. All the survivors in the fulminant NEC group underwent surgery. Those in the fulminant NEC group were more likely to have the following clinical features: sepsis preceding NEC (*P*<0.001), abdominal distention (*P*<0.001), bowel sound disappearance (*P* = 0.001), leukopenia or neutropenia (*P*<0.001), C-reactive protein (CRP) <10 mg/L (*P* = 0.003), procalcitonin (PCT) < 2 μg/L (*P*<0.001), pH ≤7.2 (*P*<0.001), and radiographic evidence of pneumoperitoneum (*P*<0.001) or seroperitoneum on ultrasonography (*P* = 0.017). In conclusion, fulminant NEC is characterized by urgent onset and prompt deterioration, potentially resulting in death. The lack of unique characteristics makes it difficult to recognize by medical caregivers. Close observation, early detection and timely surgical intervention may improve the prognosis.

## Introduction

Necrotizing enterocolitis (NEC) is the most common and serious acquired gastrointestinal emergency disease in newborn infants, with clinically significant long- and short-term consequences. With the great advances in neonatal intensive care management, an increasing number of extremely and very low birth weight infants who are highly susceptible to NEC are surviving. The overall incidence is 1–3 cases per 1000 live births, whereas the incidence is 5% in very preterm (<32 weeks’ gestational age) and very low birth weight (VLBW) infants and 10% in extremely preterm (<28 weeks’ gestational age) and extremely low birth weight (ELBW) infants[1–3]. Among these infants, a minority of cases are fulminant and mortality is rapid, with diffuse intestinal necrosis seen on laparotomy or autopsy. However, the etiology, unique characteristics and prognosis of fulminant NEC are not clear.

Few published reports have focused on fulminant cases. Hummer P *et al*. reported 42 newborns with NEC (Bell stage ≥Ⅲ) in Germany between 1969–1985; 33.3% (14/42) of them suffering from fulminant courses[4]. Voss M *et al*. studied 128 surgically treated patients with NEC in South Africa over two years. The 52 patients in the fulminant NEC group had a median gestational age of 31 weeks and birth weight of 1444 g, and 56% (29/52) of them died[5]. Lambert DK *et al*. investigated 271,327 newborns in a multihospital healthcare system in America over a 9-year period, and fulminant NEC (Bell stage ≥Ⅱ) was observed in 35 newborns, of whom the average birth weight and gestational age were 1089±545 g and 27.5±2.4 weeks, respectively. The mortality rate of fulminant NEC was 13/100,000[6].

We conducted a retrospective analysis of fulminant NEC patients admitted to the Children’s Hospital of Chongqing Medical University during the last 10 years (2007–2017) to explore the risk factors, identify the clinical characteristics and analyze the interventions and prognoses.

As the center of the Neonatal Emergency Transport System (NETS) in southwestern China, the Children’s Hospital of Chongqing Medical University serves a large geographical area, including Chongqing, Guizhou and Yunnan; from 2007 to 2017, it received 4828 to 9125 neonates annually. However, most of the neonates admitted to this center were late preterm or term infants. The constituent ratio of VLBW infants was 1.53% (74/4828) to 2.19% (200/9125), and the constituent ratio of ELBW infants was 0.08% (4/4828) to 0.33% (30/9125) during the 10-year period. Therefore, the retrospective analysis of neonates with fulminant NEC in this center over the study period might reflect the characteristics of fulminant NEC in late preterm or term infants in southwestern China.

## Patients and methods

The medical records of neonates who were diagnosed with NEC (Bell stage ≥ΠB) at the Children’s Hospital of Chongqing Medical University from 1 November 2007 to 31 October 2017 were collected. Patients were excluded if they had NEC after intestinal surgery or if they had surgical emergency conditions other than NEC (such as intestinal volvulus, congenital malrotation of intestine, acute appendicitis or meconium peritonitis). Those without complete general demographic records were also excluded. ‘Fulminant NEC’ was defined as NEC with a rapid clinical progression, with 48 h or less from the onset of symptoms (such as emesis, abdominal distension or gross bloody stool) to death or severe disease requiring surgical management[4–7]. Finally, 352 newborns were enrolled in this study, among which 112 (31.8%) fulfilled the diagnosis criteria for fulminant NEC. The data, including sex, gestational age, birth weight, risk factors, clinical manifestations, laboratory and imaging investigations, interventions and prognosis, were then recorded. Ethics approval for this study was obtained from the Institutional Review Board of the Children’s Hospital, Chongqing Medical University (Approval No. 2019–27). The need for written informed consent from the patients or their legal guardians was waived by the ethics committee because this was a retrospective study, and the data were collected and interpreted anonymously.

## Statistical analysis

All data were analyzed using *SPSS* 19.0 software for Windows. Measurement data were reported as the mean *± standard deviation* or median (*interquartile range*). Count data were reported as numbers of cases. The *Kolmogorov-Smirnov* test for normality was conducted. The measurement data were analyzed using a *t*-test if the data were normally distributed; otherwise, the *Mann-Whitney U* test was adopted. Count data were analyzed using the *chi-squared* test. *P* values less than 0.05 were considered statistically significant.

## Results

During the 10-year investigation period, 352 newborns developed Bell stage ≥ΠB NEC, of which 112 (31.8%) cases fulfilled the definition of fulminant NEC. More patients had surgical or radiological evidence of perforation in the fulminant NEC group than in the non-fulminant NEC group (62.5% *vs* 41.25%, *P*<0.001). Most of the infants enrolled in this study were late preterm or term infants. The mean gestational ages in the fulminant and non-fulminant NEC groups were 37.07 weeks and 37.21 weeks (*P* = 0.369), and the birth weights were 2610 g and 2700 g (*P* = 0.608), respectively. In the fulminant NEC group, 53.6% (60/112) were term infants and 35.7% (40/112) were late preterm infants, whereas only 10.7% (12/112) of the infants had gestational ages less than 32 weeks. Fulminant cases were more likely to present sepsis preceding the NEC diagnosis (62.5% *vs* 41.67%, *P*<0.001); however, there was no difference in antibiotic exposure between the two groups (41.96% *vs* 32.50%, *P* = 0.084) (Table 1).

**Table 1:** Demographic features and risk factors of the NEC neonates.

	Fulminant NEC	Non-fulminant NEC	*χχ*^*2*^(*Z*)	*P* value
	(n = 112)	(n = 240)		
Male gender	66(58.93%)	138(57.50%)	0.064	0.800
Gestation age (weeks), mean(P25-P75)	37.07(34.18–38.86)	37.21(34.4–39.29)	(*Z* = 0.899)	0.369
Birth weight (g), mean(P25-P75)	2610(2000–3187.5)	2700(2000–3297.5)	(*Z* = 0.513)	0.608
Cesarean section	63(56.25%)	145(60.42%)	0.548	0.459
NRDS	10(8.93%)	19(7.92%)	0.103	0.748
Sepsis	70(62.50%)	100(41.67%)	13.273	<0.001
Hypoglycemia	16(14.29%)	24(10.00%)	1.393	0.238
PDA	22(19.64%)	36(15.00%)	1.196	0.274
Anemia	56(50.00%)	109(45.42%)	0.644	0.422
Gastrointestinal tract anomalies	15(13.97%)	20(8.33%)	2.183	0.140
Ventilatory support	19(16.96%)	32(13.33%)	0.813	0.367
Exchange transfusion	4(3.57%)	3(1.25%)	1.088	0.297
Formula feeding	71(63.29%)	164(68.33%)	0.840	0.359
Antibiotic before NEC	47(41.96%)	78(32.50%)	2.987	0.084

*Abbreviations: NEC, necrotizing enterocolitis; NRDS, neonatal respiratory distress syndrome; PDA, patent ductus arteriosus.

The incidence of poor prognosis (died or withdrawn further treatment) was significantly higher in the fulminant NEC group than in the non-fulminant NEC group (62.5% *vs* 36.67%, *P*<0.001). There were 70 infants in the fulminant NEC group with a poor prognosis, of whom 10 died. Among the other 60 infants for whom treatment was terminated, 35 guardians of patients refused surgery and further treatment, 11 patients only underwent laparotomy for diffuse bowel necrosis, and 14 guardians of patients refused further treatment, such as ventilatory support, due to a serious peri-operation condition.

As summarized in Table 2, after the NEC diagnosis, the patients in the fulminant NEC group had more severe clinical manifestations than those in the non-fulminant NEC group. A higher incidence of bowel sound disappearance was observed in the fulminant NEC group (43.75% *vs* 25.42%, *P* = 0.001), whereas diminished bowel sounds occurred more often in the non-fulminant NEC group (36.61% *vs* 50.83%, *P* = 0.013). The fulminant NEC group was more likely to have abdominal distension than the non-fulminant NEC group (95.54% *vs* 81.67%, *P*<0.001). However, there was no difference between the fulminant and non-fulminant NEC groups in emesis or gross bloody stools.

**Table 2:** Clinical features of the NEC neonates.

	Fulminant NEC	Non-fulminant NEC	χ*χχ* ^*2*^(*Z*)	*P* value
	(n = 112)	(n = 240)		
Age of onset(days), mean(P25-P75)	5.54(2.00–10.81)	7.15(2.02–13.79)	(*Z* = 0.736)	0.462
Emesis	51(45.54%)	130(54.17%)	2.277	0.131
Abdominal distension	107(95.54%)	196(81.67%)	12.258	<0.001
Gross bloody stool	45(40.18%)	101(42.08%)	0.114	0.735
Bowel sound				
Attenuation	41(36.61%)	122(50.83%)	6.216	0.013
Disappearance	49(43.75%)	61(25.42%)	11.947	0.001
Complete blood count				
Leukocytes <5*10^9^/L	42(38.53%)	32(13.73%)	26.933	<0.001
Leukocytes >20*10^9^/L	5(4.63%)	34(14.66%)	7.294	0.007
Platelet ≤100*10^9^/L	23(21.10%)	38(16.31%)	1.164	0.281
Hematocrit<22.0%	5(4.59%)	10(4.29%)	0.000	1.000
Neutrophils<1.5*10^9^/L	19(17.43%)	7(3.00%)	22.004	<0.001
Unknown	3	7		
C-reactive protein≥10 mg/L	69(64.49%)	108(47.37%)	8.563	0.003
Unknown	5	12		
Procalcitonin ≥2 ¶g/L	48(84.21%)	78(53.79%)	16.131	<0.001
Unknown	55	95		
pH≤7.2	23(29.87%)	14(9.09%)	16.477	<0.001
Unknown	35	86		
Positive blood culture	19(26.76%)	32(21.33%)	0.800	0.371
Unknown	41	90		
Abdominal radiography				
Portal venous air	26(24.30%)	51(21.43%)	0.351	0.554
Pneumatosis intestinalis	42(39.25%)	83(34.87%)	0.612	0.434
Pneumoperitoneum	73(68.22%)	97(40.76%)	22.282	<0.001
Unknown	5	2		
Abdominal ultrasonography				
Portal venous air	20(37.74%)	86(46.24%)	1.208	0.272
Pneumatosis intestinalis	11(20.75%)	30(16.13%)	0.621	0.431
Seroperitoneum	45(84.91%)	127(68.28%)	5.652	0.017
Unknown	59	54		

Complete blood count results were obtained for 109 of 112 infants with fulminant NEC and 233 of 240 infants with non-fulminant NEC. The infants who developed fulminant NEC had a higher incidence of leukocytes less than 5*10^9^/L (38.53% *vs* 13.73%, *P*<0.001) and neutrophils less than 1.5*10^9^/L than those who developed non-fulminant NEC (17.43% *vs* 3.0%, *P*<0.001). However, more neonates had leukocytes greater than 20*10^9^/L in the non-fulminant NEC group (*P* = 0.007). The fulminant NEC group had more infants with C-reactive protein (CRP) ≥10 mg/L and procalcitonin (PCT) ≥2 μg/L than the non-fulminant NEC group. The average positive rate for blood culture was 23.07% (51/221) for patients with NEC in this study; more patients with a fulminant presentation had a positive blood culture than patients without a fulminant presentation, although the difference was not statistically significant (26.76% *vs* 21.33%, *P* = 0.371).

The fulminant NEC group was significantly more acidotic than the non-fulminant NEC group, and the incidence of pH ≤7.2 was higher in the fulminant NEC group (29.87% *vs* 9.09%, *P*<0.001).

A comparison of the radiological findings between the fulminant and non-fulminant NEC groups was performed. Pneumoperitoneum was observed in 68.22% of the fulminant cases and in 40.76% of the non-fulminant cases (*P*<0.001). Abdominal ultrasonography was available for 53 patients in the fulminant NEC group and 186 patients in the non-fulminant NEC group. More patients in the fulminant NEC group had seroperitoneum (84.91% *vs* 68.28%, *P* = 0.017). There was no significant difference in the incidence of portal venous air or pneumatosis intestinalis.

Surgical records were available for 73 patients with fulminant courses; most of the patients (69.86%) were found to have undergone laparotomy and enterostomy, and there was no difference compared with the non-fulminant NEC cases. The lengths of necrotic intestine and remaining intact intestine in all patients who underwent surgical interventions were recorded, and there were no significant differences between the two groups (*P* = 0.588, *P* = 0.301). In the fulminant NEC group, 42 patients survived, and all of them underwent surgery (Table 3).

**Table 3:** Surgical findings of the NEC neonates.

NEC (n)	Length of remaining intact intestine (cm)	Length of necrotic intestine (cm)	Surgery			
			Laparotomy (n, %)	Laparotomy+ Enterostomy (n, %)	Laparotomy+Anastomosis (n, %)	Laparotomy+ Repair of perforation (n, %)
Fulminant NEC (73)	84.34±3.60	31.21±4.30	13(17.81)	51(69.86)	7(9.59)	2(2.74)
Non-fulminant NEC (97)	79.32±3.21	28.17±3.62	23(23.71)	60(61.86)	13(13.40)	1(1.03)
*χ*^*2*^(*t*)	(*t* = 1.037)	(*t* = 0.543)	0.870	1.179	0.583	0.702
*P* value	0.301	0.588	0.351	0.278	0.445	0.402

## Discussion

Despite advances in neonatal medicine, NEC remains one of the common causes of morbidity and mortality in patients admitted to the neonatal intensive care unit. Infants with fulminant NEC may present specific challenges, including physiologic instability, multifocal disease and widespread peritoneal contamination, with high mortality[8]. In this study, approximately 31.81% (112/352) of the infants who developed NEC (Bell stage ≥ΠB) had rapidly fatal courses, whereas 37.50% (42/112) survived.

Instead of a single pathogenic entity, NEC is a phenotype resulting from several different pathways[9, 10]. The etiologic factors are multifactorial and largely related to the immaturity of the gastrointestinal tract[11]. The gastrointestinal tracts of preterm infants are less mature and more susceptible than those of term infants. Lambert DK *et al*. reported 105 neonates who developed NEC; the most significant associations with fulminant NEC were birth weight and gestational age. In that study, the fulminant NEC group had lower birth weights (1089±545 *vs* 1652±817 g) and earlier gestational ages (27.5±3.4 *vs* 31.1±4.4 weeks) than the non-fulminant NEC group[6]. However, in this study, it was found that birth weight and gestational age were not associated with fulminant NEC. During the last 10 years (2007–2017), the Children’s hospital of Chongqing Medical University received 79,949 neonates, among whom only 2.18% (1,741/79,949) were infants with birth weights less than 1500 g. In this series, the majority of admitted neonates (77.8%) were late preterm or term infants, and 90.3% (318/352) of the infants had birth weights greater than 1500 g. Moreover, one published report also showed that the average gestational age and birth weight of 70 neonates with NEC perforation admitted from May 1996 to August 2016 in our hospital were 36.55±3.18 weeks and 2783.46±833.09 g, respectively[12]. Therefore, it failed to explore the significant difference between the two groups in terms of gestational age and birth weight.

In previous studies, neonatal respiratory distress syndrome (NRDS), ventilatory support, patent ductus arteriosus (PDA), formula feeding, sepsis and antibiotic exposure were all reported as risk factors for NEC, especially in preterm infants[13, 14]. According to this study, 77.8% of admitted infants were late preterm or term infants, and NRDS, ventilatory support and PDA were not found to be associated with fulminant NEC. Hammond *et al*. reported that a switch from human milk to formula in preterm infants (median gestational age and birth weight were 29 weeks and 1200 g, respectively) often preceded a fulminant NEC diagnosis[7]. However, in this series, the feeding regimes between the two groups were not observed to be different. It is still not clear whether these factors, which have been previously reported, are associated with fulminant NEC in late preterm and term infants.

In this study, sepsis preceding an NEC diagnosis was more often diagnosed in the fulminant NEC group than in the non-fulminant NEC group. The infection might be closely associated with fulminant NEC. However, the specific role of infection in NEC pathogenesis is unclear; NEC might be the result of pathogen infection, abnormal intestinal bacterial colonization or incomplete epithelial barrier function, all leading to a common mechanism of bowel injury, intestinal inflammation and the release of proinflammatory mediators, initiating an inflammatory cascade causing intestinal damage and necrosis[15]. In previous studies, empirical antibiotic was also described as a potential contributing factor in the development of NEC, especially prolonged antibiotic exposure for more than 10 days, which was reported to be associated with a nearly two-fold or three-fold increase in the risk of developing NEC[16–18]. However, no difference in antibiotic exposure preceding the NEC diagnosis between the fulminant and non-fulminant NEC groups in this study was observed, indicating that antibiotic exposure did not increase the risk of developing fulminant NEC. Therefore, for patients with sepsis, timely and effective anti-infection therapy is necessary.

After an NEC diagnosis, more severe infections were observed in the fulminant NEC group than in the non-fulminant NEC group. As the gold standard of bloodstream infection diagnosis, blood culture was performed for 62.78% (221/352) of the patients with NEC, and ‘culture-proven sepsis’ was found more often in NEC patients, particularly in the fulminant NEC group. In our hospital, the average positive rate for blood culture is 5.40% overall and 7.63% in newborn patients. However, the positive rate for blood culture in NEC infants was 23.07% in this study; this result was much higher than the average positive rate, indicating that NEC often complicated severe infections especially culture-confirmed bacterial sepsis. In addition, more patients in the fulminant NEC group had a positive blood culture than those in the non-fulminant NEC group; the positive rate reached 26.76%, indicating that the fulminant cases were more vulnerable to serious infections than the non-fulminant cases. However, the difference between the two groups was not statistically significant, which might be caused by the limited number of cases and the low sensitivity of blood culture attributable to multiple factors, such as the appropriate ordering of blood cultures, timing of blood collection, sample site and impact of the volume sampled[19]. Other inflammatory indicators, including complete blood counts, CRP and PCT, could also be useful in the timely diagnosis of sepsis, helping in the differential diagnosis of non-infectious diseases. Especially in neonates with CRP ≥ 10 mg/L or PCT ≥ 2 μg/L, the possibility of progression to severe sepsis is usually considered high[20–22]. Kling PJ *et al*. reported that neutropenia was observed in severe NEC, whereas neutrophilia was observed in less-severe NEC as a normal adaptive response to inflammation[23]. Neutropenia was also associated with a poor prognosis[24]. In this study, low leukocyte counts and low neutrophil counts were observed among patients with fulminant NEC, indicating that the fulminant cases were more serious than the non-fulminant cases. These findings indicated that for NEC patients, the complete blood count, CRP and PCT should be closely followed. Usually, fulminant NEC patients are characterized by obvious hematological abnormalities.

As the most serious acquired gastrointestinal emergent disease, NEC is characterized by abdominal distension, emesis and bloody stools. Compared with the non-fulminant NEC group, the fulminant NEC group had more severe imaging findings and clinical manifestations. Higher frequencies of bowel sound disappearance and abdominal distension were observed in the fulminant NEC group. On abdominal radiography, pneumoperitoneum was more common in those who with fulminant courses than those with non-fulminant courses, while seroperitoneum was more often found on abdominal ultrasonography in the fulminant NEC group. These findings might indicate that more intestinal perforations or peritonitis occurred in the fulminant cases than in the non-fulminant cases, which conformed with the surgical findings. Furthermore, portal venous air was considered to be associated with extensive intestinal necrosis in a previous study[5]. Lambert DK *et al*. reported a multicenter, historical cohort study that involved patients with NEC (Bell stage ≥Π). They reported that more patients had portal venous air on abdominal radiography in the fulminant NEC group than in the non-fulminant NEC group[6]. However, in this study, there was no significant difference in the incidence of portal venous air on abdominal radiography between the two groups. The differences in the lengths of necrotic intestine and remaining intact intestine between the two groups did not reach statistical significance. On the one hand, this study included neonates with Bell stage ≥ΠB NEC. In the non-fulminant NEC group, the incidence of portal venous air on abdominal radiography was 21.43%, and the average length of necrotic intestine reached 28.17 cm, indicating that the infants were in serious condition. Moreover, the course of disease in the fulminant NEC group was less than 48 h, which was much shorter than that in the non-fulminant NEC group, indicating that the fulminant patients had serious conditions similar to the non-fulminant patients but with a shorter duration. In addition, all the surviving neonates in the fulminant NEC group underwent surgery in this study. This indicates that timely surgical intervention may improve the prognosis of patients with fulminant NEC.

## Conclusion

Fulminant NEC is characterized by urgent onset and prompt deterioration, potentially resulting in death. The lack of unique characteristics makes it difficult to recognize by medical caregivers. Close observation, early diagnosis and timely surgical intervention may improve the prognosis.
